# Successful induction of remission with azacitidine, venetoclax, and dasatinib followed by allogeneic hematopoietic stem cell transplantation in a patient with Philadelphia chromosome-positive mixed phenotype acute leukemia (B/myeloid): a case report

**DOI:** 10.3389/fmed.2026.1788083

**Published:** 2026-09-01

**Authors:** Juan Zhang, Yan Huang, Liangkui Luo, Kaikai Huang, Yanbin Pang

**Affiliations:** 1 Department of Hematology, Shenzhen Hospital of Southern Medical University, Shenzhen, China; 2 Department of Hematology, The Shenzhen Integrated Traditional Chinese and Western Medicine Hospital, Shenzhen, China

**Keywords:** azacitidine, case report, dasatinib, Ph^+^ MPAL, venetoclax

## Abstract

**Background:**

Philadelphia chromosome-positive mixed phenotype acute leukemia (Ph^+^ MPAL) is a rare and aggressive hematologic malignancy. The incorporation of tyrosine kinase inhibitors (TKIs) into acute lymphoblastic leukemia (ALL)-like regimens has improved survival for Ph^+^ MPAL. However, the benefit of ALL-like chemotherapy backbones for adult patients, particularly those with bilineal disease, appears limited and is often hampered by significant toxicity. To the best of our knowledge, this case adds to the limited clinical experience regarding the use of azacitidine, venetoclax, and dasatinib in Ph^+^ MPAL. Novel, safer strategies are urgently needed.

**Case presentation:**

A 32-year-old man presented with a one-week history of fever. Bone marrow examination revealed 83% blasts with two distinct populations: lymphoblasts and monoblasts, confirming bilineal B/myeloid MPAL. Karyotyping showed 45, XY,−7, t(9;22)(q34;q11.2), and the *BCR::ABL* (p190) fusion gene was positive. Molecular genetics identified an EVI1 fusion gene and an ASXL1 mutation. The patient received one cycle of induction therapy with azacitidine, venetoclax, and dasatinib, augmented with Vincristine, prednisone, and homoharringtonine. Complete remission was achieved following this cycle, with the primary adverse event being grade IV myelosuppression, managed without severe infection, tumor lysis syndrome or organ dysfunction. After three consolidation cycles, flow cytometry showed negative minimal residual disease (MRD); however, *BCR::ABL1* remained detectable at 0.3289%. The patient subsequently underwent haploidentical hematopoietic stem cell transplantation (haplo-HSCT). At the most recent follow-up, *BCR::ABL1* was undetectable.

**Conclusion:**

An induction regimen based on azacitidine, venetoclax, and dasatinib may represent a safe and effective therapeutic strategy for adult patients with Ph^+^, bilineal MPAL, particularly when augmented with agents targeting distinct lineage components.

## Introduction

Mixed-phenotype acute leukemia (MPAL), particularly the bilineal subtype, is a rare and aggressive malignancy characterized by blasts that co-express markers of more than one hematopoietic lineage. Patients with MPAL have a poor prognosis, and allogeneic hematopoietic stem cell transplantation (allo-HSCT) is often pursued after achieving remission (1, 2). There is no universally standard induction regimen, though retrospective data suggest that ALL-type regimens are generally superior to AML-type or combined approaches for many MPAL subtypes (3). However, the bilineal variant, in which distinct blast populations express different lineage markers, represents a particularly challenging subtype. In the large international iBFM-AMBI2012 study, bilineal MPAL was associated with lower complete remission (CR) rates and inferior 5-year event-free survival compared to bilineal MPAL, and the benefit from ALL-type therapy was less pronounced for this subgroup (4). This suggests that standard ALL-directed approaches may be suboptimal for bilineal disease, and hybrid regimens may offer a benefit, albeit with increased toxicity (5).

The Philadelphia chromosome (Ph), resulting from the t(9;22) (q34;q11) translocation and generating the BCR::ABL1 fusion gene, is the most frequent cytogenetic abnormality in MPAL, occurring in 20%−40% of cases (6). For bilineal Ph^+^ MPAL, standard ALL-directed chemotherapy combined with TKIs carries substantial toxicity and may be suboptimal for the myeloid component. Preclinical data suggest synergistic activity between BCL-2 inhibitors (venetoclax), hypomethylating agents (azacitidine), and BCR-ABL TKIs. This provides a biological rationale for a lower-intensity, multi-targeted induction strategy that can bridge patients to potentially curative allo-HSCT (7). Despite this progress, no standard induction regimen exists for Ph^+^ MPAL, and the optimal treatment strategy remains debated, particularly for the bilineal subtype (8). Conventional intensive chemotherapy combined with TKIs can improve outcomes but carries substantial toxicity, limiting its use in older or medically frail patients (9). This has prompted interest in novel, less toxic targeted combinations. Preclinical and retrospective data suggest synergistic activity between BCL-2 inhibitors (e.g., venetoclax) and BCR::ABL1 TKIs (10, 11). The combination of venetoclax with a hypomethylating agent (HMA) such as azacitidine is already an established standard for AML patients unfit for intensive chemotherapy. Notably, a case series by Zou JY et al. demonstrated that venetoclax, an HMA, and a TKI induced CR in four patients with Ph^+^ MPAL (12). However, the efficacy of such venetoclax-based regimens specifically for the bilineal B/myeloid subtype of MPAL requires further validation.

Here, we report a case of bilineal B/myeloid Ph^+^ MPAL with a lymphoblastic and monocytic immunophenotype. The patient achieved CR with an induction regimen that combined venetoclax, azacitidine, homoharringtonine and dasatinib, along with Vincristine and prednisone. This case adds to the limited evidence supporting venetoclax-based, TKI-containing strategies for this high-risk entity and underscores the need for further investigation.

## Case report

A 32-year-old man presented to Shenzhen Hospital, Southern Medical University on Day 1 (All calendar dates have been converted to relative time points), with a 1-week history of fever. He had no prior medical history of hypertension, diabetes mellitus, chronic hepatic or renal disease, or any hematologic disorder. He denied smoking, alcohol abuse, or occupational toxin exposure, and his family history was unremarkable for hematologic or other malignancies. No previous chemotherapy, radiotherapy, or immunosuppressive therapy had been administered. Physical examination revealed an anemic appearance without palpable hepatosplenomegaly or lymphadenopathy. Initial laboratory findings showed marked leukocytosis (white blood cell count 59.98 × 10^9^/L), normocytic anemia (hemoglobin 115 g/L), and severe thrombocytopenia (platelet count 37 × 10^9^/L).

## Morphology

**Figure 1:**
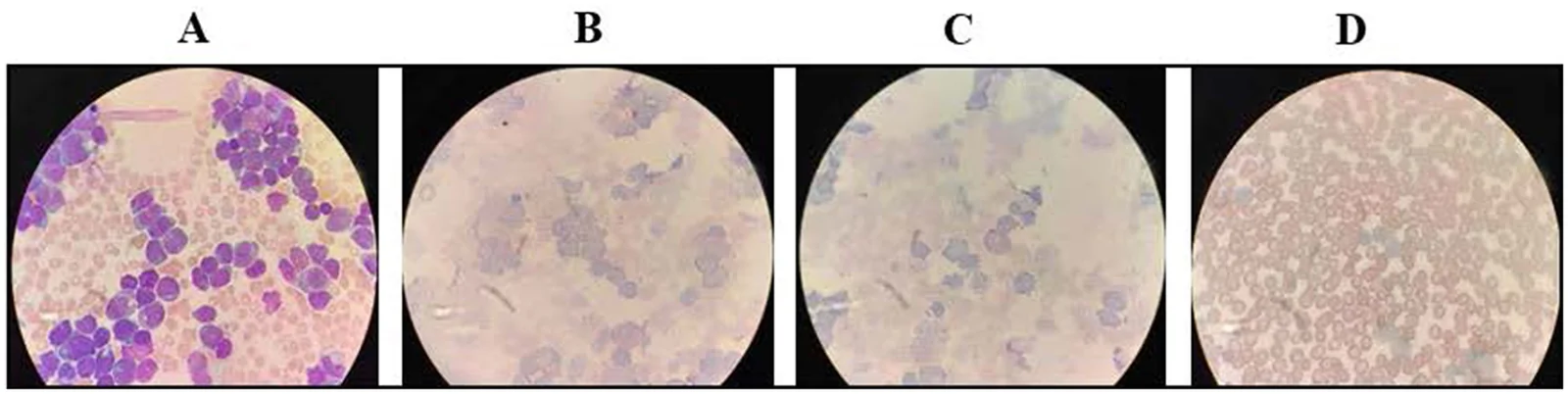
Illustration of the bone marrow morphology characteristics. The marrow is markedly hypercellular (hyperplastic). Two distinct populations of blast cells are identified: **(A)** Bone marrow aspirate showing hypercellular marrow (H&E, × 400); **(B)** PAS-positive lymphoblasts; **(C)** POX-positive monoblasts; **(D)** NSE-positive monoblasts with sodium fluoride inhibition.

Bone marrow aspiration revealed a markedly hypercellular marrow with two distinct blast populations: population 1 (lymphoblast-like) comprised small cells with scanty cytoplasm, finely dense nuclear chromatin, and positive periodic acid–Schiff (PAS) staining; Population 2 (monoblast-like) consisted of larger cells with abundant cytoplasm, loose chromatin, and folded nuclei, positive for peroxidase (POX) and sodium fluoride–inhibitable esterase (Figure 1).

### Multiparameter flow cytometry

**Figure 2:**
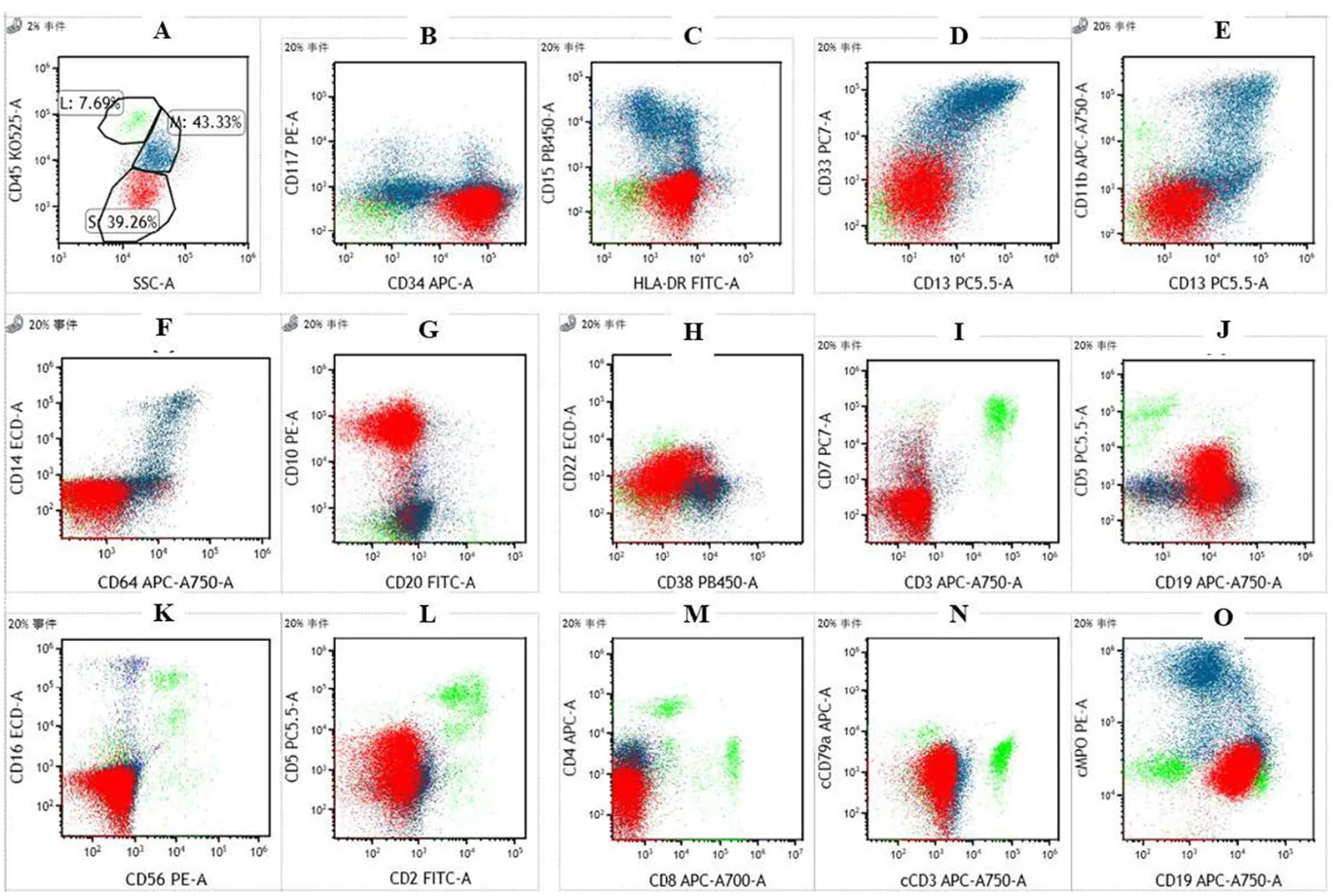
**(A–O)** Illustration of the immunophenotypic analysis by multiparameter flow cytometry, revealing two distinct populations of blast cells in the bone marrow: lymphoblasts (marked in red) and myeloid (monocytoid) blasts (marked in blue). The population of primitive B-lymphoblasts exhibits a characteristic immunophenotype: positive for CD19 and strongly positive for CD10, while negative for cytoplasmic cMPO, cytoplasmic cCD3, and other myeloid and T-lymphoid antigens. The second population presents a pre-monocytic appearance, showing a corresponding immunophenotype: strongly positive for CD33, partially positive for CD34 and CD64, and positive for CD36, CD15, and cMPO.

Multiparameter flow cytometry (eight-color, Beckman Coulter Navios) confirmed the bilineal nature. The lymphoblast population (39.26% of total events) was CD45-dim/negative, low side scatter, and expressed CD19 (bright), CD34, CD10, and HLA-DR, but was negative for cytoplasmic CD79a, surface kappa/lambda, CD20, CD33, CD13, CD117, cMPO, and cCD3. The monoblast population (43.33%) strongly expressed CD33, CD13, partial CD64, CD36, CD15, CD117, and cytoplasmic myeloperoxidase (cMPO), while being negative for CD19, cCD3, CD79a, and nuclear TdT (Figure 2). According to the WHO 2022 and International Consensus Classification (ICC) criteria, a diagnosis of B-cell/myeloid mixed-phenotype acute leukemia (MPAL) was established.

### Cytogenetics

Cytogenetic analysis was performed on GTG-banded metaphase chromosomes from bone marrow cultures, with karyotype described according to ISCN 2020. A total of 20 metaphases were analyzed, revealing a complex karyotype in 4 cells: 45, XY,-7, t(9;22)(q34;q11.2) (4). Qualitative reverse-transcription PCR for BCR::ABL1 transcripts, identified the e1a2 (p190) isoform showed positivity. No e13a2 or e14a2 (p210) transcripts were detected. Concurrently, Qualitative RT-PCR for MECOM (EVI1) transcript was positive (normalized to ABL1). Next-generation sequencing (NGS, a 341-gene myeloid panel performed on the Illumina Next-Seq platform, with a mean coverage of 844 × ) additionally identified an ASXL1 frameshift mutation, c.2077C >T (p.R693^*^12), with a variant allele frequency (VAF) of 33.3%, indicating a likely clonal mutation. No co-occurring mutations in RUNX1, TP53, NRAS, KRAS, FLT3-ITD, or IDH1/2 were detected.

The patient was diagnosed bilineal high-risk B/myeloid mixed-phenotype acute leukemia (MPAL) with Philadelphia chromosome (Ph^+^, p190 isoform), monosomy 7, and concomitant ASXL1 mutation, which was established based on the WHO 2022 and International Consensus Classification (ICC),: (i) the presence of two distinct blast populations, each accounting for >20% of total nucleated cells; (ii) lymphoblasts meeting stringent B-lineage criteria (CD19 bright, CD10 positive) and myeloblasts fulfilling the myeloperoxidase-positive myeloid lineage requirement; and (iii) exclusion of other entities such as blastic plasmacytoid dendritic cell neoplasm and acute undifferentiated leukemia. Most importantly, the patient is positive for the *BCR::ABL1* fusion gene. The presence of complex karyotype, EVI1 expression, and a myeloid-relevant ASXL1 mutation collectively portend a particularly adverse prognosis, which was communicated to the patient before initiating dasatinib-based induction chemotherapy.

Given the bilineal disease and high-risk genetic profile, a response-adapted, multi-targeted induction regimen was employed, combining lymphoid- and myeloid-directed agents with targeted therapy: Vincristine (VCR): 2 mg on days 1, 8; Prednisone (Pred): 100 mg daily, days 1–14 (then tapered); Homoharringtonine (HHT): 2 mg daily, days 1–7; Azacitidine (AZA): 100 mg daily, days 1–10; venetoclax (VEN): 100 mg day 1, 200 mg day 2, 400 mg days 3–28; dasatinib: 100 mg daily days 3–12, reduced to 50 mg daily days 13–28 due to abnormal liver function tests. Post-induction bone marrow evaluation demonstrated a hypocellular marrow with only 4% blasts, confirming morphological remission. The patient developed Grade IV neutropenia (ANC < 0.5 × 10^9^/L) lasting 18 days (from day 2 to day 20 post-induction). During the period of neutropenic, the patient developed fever and grade 2 diarrhea. The anti-infective treatment included cefoperazone-sulbactam, meropenem, piperacillin-tazobactam, vancomycin, and voriconazole, among others. Given the high tumor burden (WBC 59.98 × 109/L, 83% blasts), hydroxyurea was given to reduce leukocytosis., tumor lysis syndrome (TLS) prophylaxis was initiated prior to therapy commencement, consisting of aggressive intravenous hydration (3 L/m2/day), urine alkalinization, and allopurinol (300 mg daily). Rasburicase was not required as the patient did not develop laboratory TLS. No severe infection, organ dysfunction, or clinical TLS was observed.

Minimal residual disease (MRD) was assessed by 8-color multiparameter flow cytometry (Beckman Coulter Navios) using a panel of antibodies including CD45, CD34, CD117, CD19, CD10, CD33, CD13, CD64, CD36, CD15, cMPO, cCD79a, cCD3, and nuclear TdT. The leukemia-associated immunophenotype (LAIP) approach was used, with MRD negativity defined as < 0.01% (10^−4^) leukemic blasts among total nucleated cells, based on the absence of both LAIP-positive and aberrant phenotypic populations.

Flow cytometry showed no evidence of MRD (MRD-negative), and the real-time quantitative PCR assay showed a BCR/ABL (p190) 0.78% identified with a normalized BCR::ABL1/ABL1 ratio (detection sensitivity: 0.01%). At diagnosis, the patient was neurologically asymptomatic, and no cerebrospinal fluid (CSF) analysis was performed owing to the absence of clinical indications and the patient's acute clinical condition. However, given the significant B-lymphoblastic component of the disease, which carries a high risk of CNS involvement, intrathecal chemotherapy was incorporated into the treatment plan following the achievement of morphological remission. The patient received CNS prophylaxis with intrathecal methotrexate and cytarabine, which was well tolerated.

No HLA-identical sibling donor was available. A search for a matched unrelated donor (MUD) was initiated through the China Marrow Donor Program; however, the projected search time was 2–3 months, during which the risk of molecular progression was deemed unacceptable given the persistent BCR::ABL1 positivity and high-risk genetics. The patient's son (age 11 years) was identified as a 5/10 HLA-matched haploidentical donor (one haplotype shared, mismatched at HLA-A, -B, -C, -DRB1, and -DQB1 loci). Cord blood was selected as a co-infusion product (5/6 HLA-matched, 2 units, total nucleated cell dose 1.66 × 10^8^/kg) to enhance engraftment and potentially augment the graft-vs.-leukemia effect, as supported by prior studies in haploidentical transplantation.

**Figure 3:**
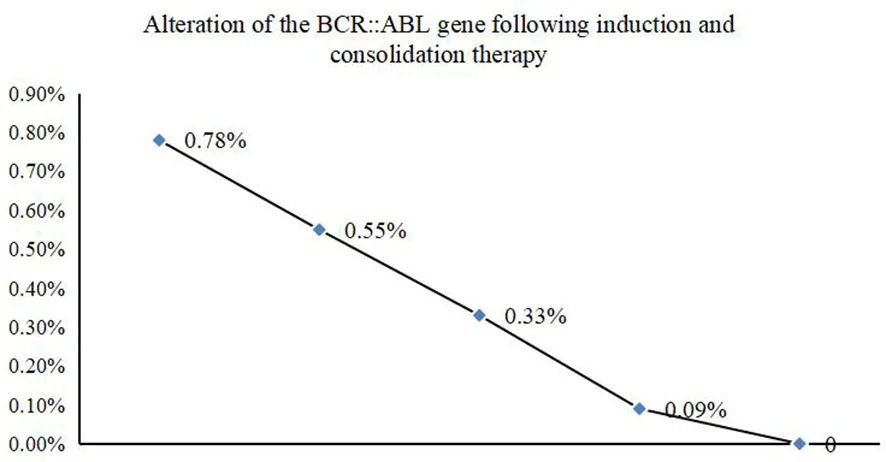
Illustration of the dynamic changes in the patient's BCR::ABL transcript levels throughout the treatment course. Following induction and consolidation therapies, the BCR::ABL level demonstrated a gradual decline. Ultimately, the patient achieved complete molecular negativity after undergoing allogeneic hematopoietic stem cell transplantation, indicating a deep molecular response.

**Figure 4:**
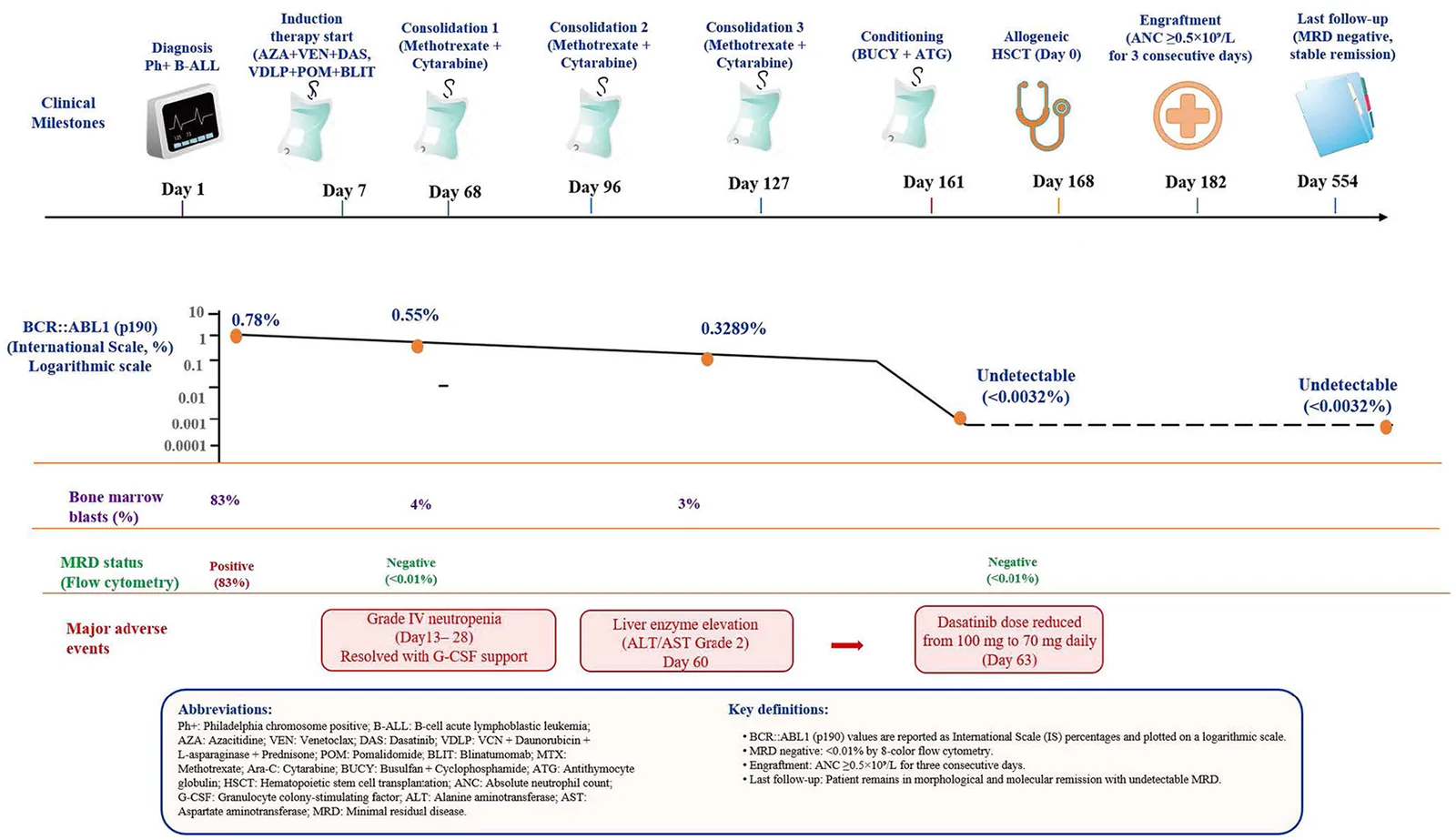
Clinical timeline illustrating treatment course, molecular response, and transplantation outcome. A horizontal timeline summarizes the patient's clinical course from Day 1 to Day 539. Key clinical milestones include the initial diagnosis of Philadelphia chromosome-positive B-cell acute lymphoblastic leukemia (Ph+ B-ALL), initiation of induction therapy with azacitidine, venetoclax, dasatinib, VDLP, pomalidomide, and blinatumomab, three sequential consolidation cycles, commencement of conditioning chemotherapy, allogeneic hematopoietic stem cell transplantation (allo-HSCT), neutrophil engraftment, and the last follow-up. Serial BCR::ABL1 (p190) transcript levels (International Scale, IS) are plotted on a logarithmic scale, demonstrating a progressive molecular response from 0.78% to 0.55%, 0.3289%, and finally undetectable after transplantation. Bone marrow blast percentages decreased from 83% at diagnosis to 4% after induction therapy and 3% before transplantation. Minimal residual disease (MRD), assessed by multiparameter flow cytometry, converted from positive at diagnosis to negative after induction and remained negative before transplantation. Major treatment-related adverse events included Grade IV neutropenia, which resolved with granulocyte colony-stimulating factor (G-CSF) support, and Grade 2 liver enzyme elevation, which prompted a reduction of the dasatinib dose while allowing continuation of therapy. At the last follow-up, the patient remained in sustained hematologic and molecular remission with undetectable MRD. AZA, azacitidine; VEN, venetoclax; DAS, dasatinib; VDLP, vincristine, daunorubicin, L-asparaginase, and prednisone; POM, pomalidomide; BLIN, blinatumomab; MTX, methotrexate; Ara-C, cytarabine; BUCY, busulfan plus cyclophosphamide; ATG, antithymocyte globulin; allo-HSCT, allogeneic hematopoietic stem cell transplantation; MRD, minimal residual disease; ANC, absolute neutrophil count; G-CSF, granulocyte colony-stimulating factor; IS, International Scale.

The patient underwent three cycles of consolidation chemotherapy: 1st Consolidation (Day 38): repeated VP+HAV regimen with dasatinib (100 mg daily). *BCR::ABL1* decreased to 0.55% (Day 64). 2nd Consolidation (Day 68): Methotrexate+ Cytarabine combination. *BCR::ABL1* further decreased to 0.3289% (Day 98). 3rd Consolidation (CAM regimen, starting Day 100): Cyclophosphamide (1,600 mg days 1, 8), Cytarabine (180 mg days 1–3, 8–10), and 6-Mercaptopurine (100 mg days 1–7). Pre-transplant assessment (Day 131) bone marrow morphology showed 3% blasts, and flow MRD remained negative. However, the *BCR::ABL* transcript level persisted at 0.3289% with qRT-PCR despite three cycles of consolidation. This persistent molecular positivity, coupled with the patient's high-risk genetic features (ASXL1 mutation, EVI1 expression, and monosomy 7), indicated a high risk of relapse. Given that further chemotherapy was unlikely to achieve molecular clearance and could add toxicity without guarantee of deeper response, the decision was made to proceed directly to allo-HSCT. No HLA-matched sibling donor was available. A search for an unrelated donor was initiated but would have taken 2–3 months, during which the patient's disease could have progressed. Given the availability of a healthy, haploidentical son (O → O) who was a 5/10 match, we opted for a haploidentical transplant with cord blood co-infusion to potentially enhance engraftment and graft-versus-leukemia effect. This approach allowed for timely intervention. Conditioning began on Day 131 (day−9), consisting of busulfan (3.2 mg/kg/d−7 to−4), cyclophosphamide (60mg/kg/d−3 to−2), and Semustine (450 mg day−3). GvHD prophylaxis included anti-thymocyte globulin (ATG), Cyclosporine, Mycophenolate Mofetil (MMF), and short-course Methotrexate (15 mg day +1; 10 mg days +3 and +6). Transplantation was performed on day 0 (Day 138), using peripheral blood stem cells from a haploidentical donor (son to father, O → O) co-infused with umbilical cord blood. Cell doses were: peripheral CD34^+^ 8.85 × 106/kg and MNC 5.62 × 108/kg; cord blood CD34^+^ 0.99 × 106/kg and MNC 1.66 × 108/kg. Neutrophil and megakaryocytic engraftment occurred on day +12. At the last follow-up on Day 538, the patient remained in excellent clinical condition with sustained molecular remission, achieving a BCR/ABL (p190) transcript undetectable identified with a normalized BCR::ABL1 ratio (detection sensitivity: 0.01%) (Figure 3) (Quantitative *BCR::ABL1* monitoring was performed by qRT-PCR with ABL1 normalization on an IS scale. The reported value of ‘0%' represents undetectable transcript below the assay's quantitative range. The detection sensitivity is 0.01% IS (corresponding to 4.5 log reduction from a standardized baseline). The complete treatment timeline is summarized in Figure 4.

## Discussion

This case report describes the successful management of a young adult with high-risk, bilineal Ph^+^ B/myeloid MPAL using a novel, targeted therapy-based induction regimen followed by haploidentical HSCT. Our discussion focuses on the key clinical and biological decisions, contextualizing them within the existing literature.

A prominent feature of this case was the presence of the Philadelphia chromosome. The introduction of TKIs like imatinib has markedly improved the prognosis of Ph^+^ acute leukemias (13). However, the optimal backbone for combining with TKIs in Ph^+^ MPAL remains unclear. Intensive ALL-type or hybrid chemotherapy with TKIs can achieve high CR rates but is associated with significant toxicity, often preventing patients from proceeding to potentially curative HSCT (9). The selection of this induction regimen was guided by four biological and clinical considerations: (i) the bilineal nature of the disease required coverage of both lymphoid and myeloid lineages; (ii) the BCR::ABL1 fusion mandated TKI inclusion; (iii) the high tumor burden and need for rapid cytoreduction favored a venetoclax-azacitidine backbone over intensive chemotherapy; and (iv) the monocytic differentiation (CD34+/CD38- immunophenotype) raised concern for MCL-1-mediated resistance to venetoclax, prompting the addition of homoharringtonine.

In our case, we chose a less intensive but targeted approach, using a VP (Vincristine and prednisone) regimen as the lymphoid-directed component, combined with dasatinib. This strategy was informed by studies showing that dasatinib plus prednisone induction, followed by consolidation and HSCT, can yield excellent 3-year overall survival (~88%) (14). This demonstrates that for patients intended for transplant, a highly intensive induction is not an absolute prerequisite and may be unnecessarily toxic. Despite the efficacy of TKIs, some Ph^+^ leukemia patients still experience relapse. Beyond TKI resistance mutations, the overexpression of anti-apoptotic proteins like BCL-2 plays a significant role in treatment failure (15). In Ph^+^ leukemias, the *BCR::ABL1* oncoprotein upregulates BCL-2, contributing to the persistence of leukemia stem cells. Venetoclax, a BCL-2 inhibitor, exhibits synergistic effects with TKIs, effectively eradicating Ph^+^ leukemia cells in preclinical models (11, 12). The addition of azacitidine, which downregulates MCL-1 and induces Noxa, further potentiates venetoclax activity (16). This triple combination (TKI + HMA + VEN) has shown promising clinical activity in Ph^+^ acute leukemias, with an overall response rate of 88.9% in a recent case series (12). Our case aligns with these findings, as the patient achieved a rapid morphological and MRD-negative remission after one cycle.

A key biological feature of our case was the monocytic differentiation of the myeloid blast population. The response of acute monocytic leukemia to the VA (VEN+AZA) regimen can be heterogeneous, potentially related to the differentiation stage of the monocytic cells (17). Research suggests that early-stage monocytic cells are more dependent on BCL-2, while mature monocytic cells rely more on MCL-1 for survival (18). The immunophenotype of the monocytic blasts in this case (CD34^+^/CD38^+^) suggested a relatively mature stage, which might confer resistance to the VA regimen (19). To mitigate this risk, homoharringtonine (HHT) was incorporated into the induction regimen. HHT promotes the degradation of MCL-1 via ubiquitination and may counteract the anti-apoptotic effects of BCL-2 (20). Clinical trial results have shown that the VHA (VEN + HHT + AZA) regimen improves the composite complete remission rate and 1-year overall survival compared to VA alone in patients with relapsed/refractory AML (21). Therefore, the addition of HHT was a rational, biology-driven decision to specifically target the monocytic component and overcome potential resistance to the VA regimen.

The patient's high-risk genetic profile—including an ASXL1 mutation, EVI1 overexpression, and monosomy 7—collectively defined an ultra-high-risk phenotype. ASXL1 mutations are associated with poor outcomes and potential resistance to hypomethylating agents in myeloid malignancies (22); EVI1 overexpression is an independent predictor of chemotherapy resistance and early relapse in AML (23); and monosomy 7 is frequently observed in MPAL and portends inferior survival (24, 25). Rather than viewing these as isolated findings, we considered them as an integrated risk signature that expedited the transition to allo-HSCT once morphological remission was achieved, as further chemotherapy was unlikely to overcome the biological aggressiveness conferred by this triad of abnormalities.

The persistent molecular positivity (BCR::ABL1 0.3289%) after three consolidation cycles, despite flow cytometry MRD negativity, served as a critical indicator of residual disease burden. Flow cytometry MRD negativity was defined as < 0.01% (10^−4^) leukemic blasts using the LAIP approach; however, this sensitivity is inferior to qRT-PCR for BCR::ABL1 (sensitivity 0.01% IS). This discordance, combined with the high-risk genetic profile (*ASXL1* mutation, *EVI1* expression, and monosomy 7), indicated a substantial risk of molecular relapse. We therefore concluded that further chemotherapy was unlikely to achieve deeper molecular clearance and would only add toxicity without guaranteeing eradication of the Ph^+^ clone, prompting the decision to proceed directly to allo-HSCT.

Finally, the transplant strategy was tailored to the patient's urgent need and lack of a matched donor. The choice of a haploidentical donor, combined with cord blood co-infusion, allowed for rapid transplantation. The cord blood co-infusion was intended to potentially improve engraftment and reduce the risk of graft failure, a concern in haploidentical transplants (26). Post-transplant, the patient achieved full donor chimerism and sustained molecular remission, demonstrating the success of this approach.

In summary, this case illustrates a rational, biology-driven treatment paradigm for high-risk, bilineal Ph^+^ MPAL. The induction regimen, built upon the synergistic combination of VEN, AZA, and DAS, with the strategic addition of HHT to target the monocytic component, proved safe and effective. The subsequent decision to expedite haplo-HSCT was guided by a comprehensive assessment of phenotypic and molecular MRD, as well as the patient's high-risk genetic profile. Our findings underscore the importance of integrating molecular and cytogenetic data with treatment response to guide timely, individualized therapeutic decisions in this rare and aggressive leukemia subtype. Prospective studies are warranted to validate this strategy and establish standardized, evidence-based treatment protocols.

## Data Availability

The original contributions presented in the study are included in the article/supplementary material, further inquiries can be directed to the corresponding author.
